# Hand-to-Environment Contact during Indoor Activities in Senior Welfare Centers among Korean Older Adults: A Pilot Study

**DOI:** 10.3390/healthcare9020105

**Published:** 2021-01-20

**Authors:** Hyang Soon Oh, Youngran Yang, Sun Young Jeong, Mikyung Ryu

**Affiliations:** 1 Department of Nursing, College of Life Science and Natural Resources, Sunchon National University, Suncheon 57922, Korea; ohs2016@sunchon.ac.kr; 2 College of Nursing, Research Institute of Nursing Science, Jeonbuk National University, Jeonju 54896, Korea; 3 College of Nursing, Konyang University, Daejeon 35365, Korea; jsy7304@konyang.ac.kr; 4 Department of Nursing, Daegu University, Daegu 42400, Korea; ryumk@daegu.ac.kr

**Keywords:** contact, hand, environment, transmission, infection, aged

## Abstract

(1) Background: This study aimed to describe the characteristics of hand-to-environment contact (HEC) and identify the influencing factors of HEC behavior during the indoor daily life of Korean older adults in senior welfare centers. (2) Methods: A cross-sectional observational study was used with 30 participants over 65 years of age attending programs in senior welfare centers. Video recordings of the 30 participants were collected for two hours a day for participants selected from 20 November to 4 December 2018. Contact frequency, density, and duration were measured. (3) Results: Video recordings of 3,930 HEC cases were analyzed. Furniture surface (25.0%), tableware and cooking utensils (5.4%), phones (5.3%), and door handles (0.1%) were found to be the items with the most frequent contact, in this order. The average contact frequency and contact density (frequency-duration/min/person) of HEC for two hours were highest for the Category I equipment (personally used, accounting for 70.4%), and the average contact duration of HEC was highest in the Category III equipment (commonly used, 47.7 s/contact/person). Contact density was as high as 266.5 (frequency-duration/min/person). Participants above 75 years of age and the unemployed showed high HEC with Category III. (4) Conclusions: Older adults need to be educated to avoid unnecessary hand contact with items in Category III. In particular, hand hygiene and sanitization through the regular and thorough disinfection of furniture surfaces and shared equipment are very important to prevent the spread of pathogens.

## 1. Introduction

Environmental surfaces can be easily contaminated with pathogens such as bacteria, fungus, and viruses by contact, either droplet or airborne [1]. Hand contact is the most common transmission mode of these pathogens [2,3]. Indoor equipment and the indoor environments where we live and stay in every day, including homes, schools, offices, work places, and other settings, are often contaminated with potentially unsafe microorganisms in aerosol droplets or fomites [4]. Once viruses settle on environmental surfaces, they can remain transmissible for hours, days, or even up to a month [4]. Contamination of indoor environmental surfaces with a rhinovirus was reported at 35% of 150 environmental sites studied, and common virus-positive sites were door handles, pens, light switches, television remote controls, faucets, and telephones [5].

For household transmission, it was reported that at least 14 persons could be contaminated by hand contact with the same door handle, and successive transmission could spread up to the sixth contact person under everyday living conditions [6]. Therefore, hand hygiene and surface cleaning were concurrently recommended [7].

In the Republic of Korea, the aging population has grown rapidly, with the proportion of people over 65 years increasing up to 14.9% of the population in 2019. This proportion is expected to increase to 20.3% in 2025. Owing to the rapid aging of the Korean society, the proportion of the population of older adults using senior welfare centers has also been increasing rapidly [8]. In 2015, the Middle East Respiratory Syndrome (MERS) outbreak identified older adults as the risk group for infection [9]. Similarly, older adults are the most vulnerable population group with regard to the COVID-19 disease [10]. Age is one of the most important risk factors for infection. Moreover, environmental contact transmission of pathogens will make older adults more vulnerable to infection according to their increasing use of senior welfare centers. Thus, it is necessary to implement preventive measures to avoid the spread of infection in commonly used places such as senior welfare centers, where older adults spend a lot of time during the day [2,3]. As a first step, the characteristics of hand-to-environment contact (HEC) in senior welfare centers should be identified in terms of contact frequency, contact duration, and contact density [11] as a means of measuring the environmental exposure to infectious pathogens [12,13]. However, there are very few studies on HEC during general daily community life among older adults [4] and about healthcare workers or patients in healthcare facilities [14,15]. Studies on HEC among older adults in daily life have not yet been conducted in Korea.

As a pilot study, this study aims to describe the characteristics of HEC among Korean older adults’ indoor activities in senior welfare centers, identify related factors that influence their HEC behavior, and generate information for instituting preventive measures for environmental control for older adults.

## 2. Materials and Methods

### 2.1. Study Participants and Data Collection

This study was a cross-sectional observational study undertaken to quantify the contact frequency, duration, and density in the daily activities of Korean older adults in senior centers and understand the factors related to their HEC behaviors. Thirty older adults over 65 years of age consented to participate voluntarily in this study, and their activities were video-recorded to collect research data on their daily activities from 20 November to 4 December 2018. The eligibility for participation did not include requirements other than age over 65 years of age and attendance of programs in senior welfare centers. The sample size of 30 participants was determined for the pilot study by considering the participation size in previous studies that used the video observation method [11,16,17] and the budget and period of the research project.

The participants were recruited from three senior welfare centers in the capital city, south, and middle regions of Korea. Senior welfare centers in Korea provide lifelong educational/recreational opportunities through various facilities for art, music, literature, leisure, sports, and health programs that meet the welfare needs of seniors in the community. We, the research team, first contacted the directors of the selected senior welfare centers and obtained permission for recruiting participants and videotaping in the welfare center. The directors of the centers introduced the study to the older adults, and the research team explained the purpose of this study, the videotaping methods, and other details about recording their activities. We spent enough time with the older adults interested in participating in this study to answer questions and help them understand the purpose and methods of the study. They then consented to participate voluntarily. Participants were informed that after observing the activities of participants through the recorded videotapes, the research team would keep the video recordings confidential for the protection of their personal privacy. Participants were given a small financial reward for their participation in the study.

Regarding videotaping location, classrooms in the senior welfare center were selected for observing indoor activities in a daily living environment without any external interference. Videotaping was adopted as the method for observation [16,17,18], and the taping was carried out with only the video on and the audio silent. The movements of the participants were fully recorded during classes (multi-camera shooting), and the high-resolution format used enabled accurate viewing. The format also supported high-quality auto-focusing that maintains focus without losing the view of the participants’ movements. Two video cameras were installed to ensure the collection of sufficient data for analysis. The participants were aware that they were being videotaped; however, to minimize their behavior changes because of the video observations, we did not inform them that we were focusing on them touching specific environmental equipment. We collected the videotape data for the two hours at the time of the day when the senior welfare centers had the most activity, which the center directors identified and suggested. Participants performed daily routine tasks as normally as possible without being self-conscious during videotaping.

The video files were observed by trained video readers. Contact with the equipment was observed, and the duration of the contact was recorded in a standardized Excel format. The reading time to confirm contact duration was entered as the start minute, end minute, start seconds, and end seconds (referring to the time at the bottom of the video player) to avoid missing data. If the reading time was missed, it would be rechecked by video playback to confirm the actual data.

This study was approved by the Institutional Review Board from the Sunchon National University (Suncheon, Korea) (104173-201809-HR-026-04).

### 2.2. Classification of Environmental Equipment for Risk of Contact Transmission

The environmental equipment items were classified into three categories depending on the degree of sharing with others: Category I: Equipment used by the individual (equipment used personally only) (e.g., mobile phone, necklace, pen, glasses, bags, etc.), Category II: Equipment mainly used by the individual but occasionally used by other people (e.g., tissues, book, cup, etc.), and Category III: Equipment for public use (commonly used equipment) (e.g., furniture surface, door handle, posters, etc.).

### 2.3. Validity and Reliability of Video Data Reading

We recruited two readers for cross-checking the videotape readings, and they were trained three times to confirm the accuracy and reliability of video data reading. The test–retest reliability of the primary and secondary readings was measured by Pearson correlation and reliability coefficients, where the coefficient value of the contact frequency was found to be 0.527 and that of the contact duration to be 0.835. Intraclass correlation coefficients (ICCs) for inter-readers reliability were defined [19]. The ICC of the contact frequency reading was 0.72 (−0.28–0.94), and the ICC of the contact duration reading was 0.92 (0.64–0.98), thus confirming the reliability of the video readings.

### 2.4. Contact Density

We developed an indicator, contact density, to quantify the intensity of contact lasting in a given time in this study. It was defined by multiplying contact frequency (number per person) and contact duration (sum of contact minutes per person) divided by the given observation time in minutes (frequency-duration/min/person). Contact density refers to the strength of the contact reflecting the contact frequency and duration for a certain period. Contact frequency measures only the quantitative aspect of the contact for a certain period, whereas contact density includes the cumulative time of how long the contact lasted if the contact frequency is the same. Hence, contact density can be identified as the qualitative aspect of the contact. It can also be more useful to measure the exposure risk of HEC than its frequency or duration alone because it contains the exposure duration for risk assessments [12].

### 2.5. Data Analysis

Descriptive data were summarized as frequencies, mean, standard deviation, and quartile of contact duration, frequency, sum of contact duration, and contact density by subgroups of environmental equipment (i.e., Categories I, II, and III). The median value was used because the distribution of the contact density was very skewed. Differences in contact density according to the participants’ characteristics, including sex (male, female), age (66–75 years, 76–85 years), employment (employed, unemployed), household income (<2,000,000, ≥200 Korean won), education (≤middle school, ≥high school), and household size (1, ≥2), were analyzed using the median value (1Q: 3Q), and the non-parametric Mann–Whitney test among two independent samples. Statistical significance level of less than 0.05 was used. Data analysis was conducted using SPSS 21.0 (IBM Corp., Armonk, NY, USA) for Windows.

## 3. Results

### 3.1. General Characteristics of Participants

Table 1 lists the characteristics of the participants in this study. Of the participants, 50% were male aged between 76 and 85 years, 53.3% had an educational background below high school, and 40.0% lived alone.

### 3.2. Observed Equipment of Hand-to-Environment Contact by Subgroups of Environmental Equipment

A total of 30 participants were observed to have made 5467 contacts with environmental equipment during two hours (60 person-hours). Finally, 3930 cases were analyzed after 1537 cases unidentifiable for HEC were excluded due to the contact being covered by another person, inadequate information regarding the equipment classification, and contacts with more than two equipment items at the same time.

Table 2 shows the most frequently contacted equipment items observed to have the maximum HEC in two hours. Furniture surfaces such as billiard pool tables, tables, and chairs were found to have the most frequent contacts, with a total of 938 times and a mean of 31.2 times/person. These were followed by tableware and cooking utensils and then phones, including mobile phones, which were the items with the second and third most frequent contacts, respectively. The frequency of contact for the door handle was five times.

### 3.3. Descriptive Statistics of Hand-to-Environment Contact by Subgroups of Environmental Equipment

The average contact frequency of HEC in two hours was the highest for the Category I equipment, accounting for 70.4% of the contacts, followed by the Category III equipment (26.6%). Each participant had HEC with the Category III equipment for public use, with an average of 92.2 times/person in two hours. The average contact duration of HEC in two hours was the highest for the Category III equipment with median of 9.6 s/contact/person, followed by the Category I equipment with median of 9.4 s/contact/person. The average of the sum of contact duration (min/person) was 22.7 in Category I, 7.6 in Category III, and 1.0 in Category II. The median contact density (frequency-duration/min/person) was 784.5 in Category I, 266.5 in Category III, and 3.1 in Category II (Table 3).

The median, 1Q, and 3Q show that the distribution of contact frequency, contact duration, and contact density had extremely high values, such that outliers were above the third quartile.

**Figure 1:**
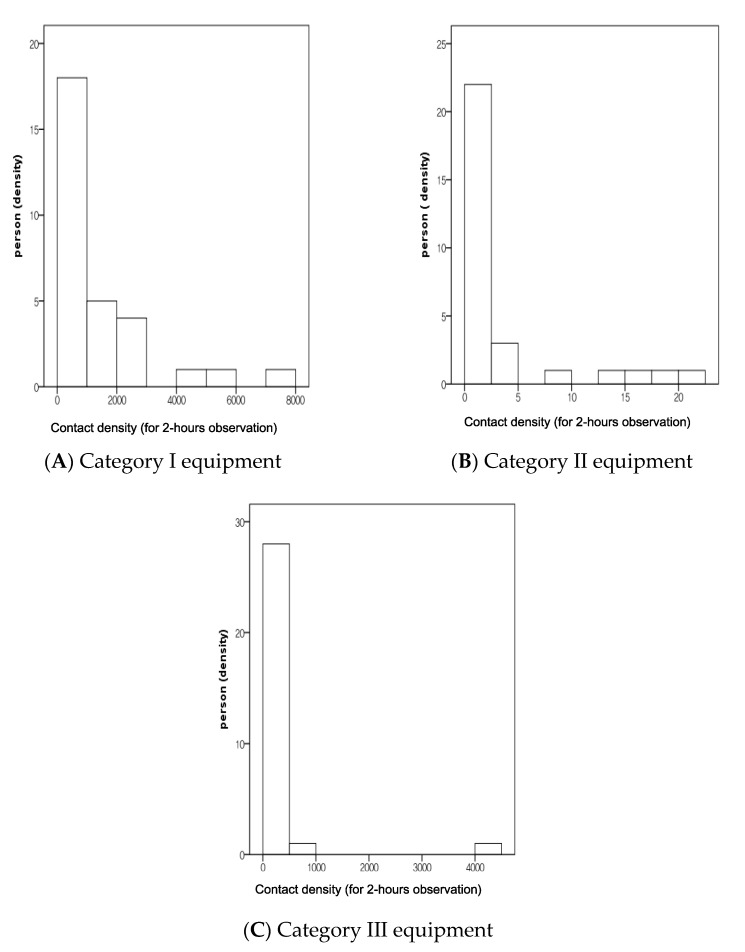
Histogram for the distribution of contact density by subgroups of equipment. Note: Y is the probability density of the frequency of a person. X is the contact density (frequency-duration/min) reflecting the contact frequency and contact duration during the 2-hours observation time. Category I: Equipment used by individuals (personally used equipment); Category II: Equipment mainly used by individuals but occasionally by other person, and Category III: Equipment used for public use (commonly used equipment).

For example, in Figure 1, the histogram for the distribution of contact density by subgroup equipment is skewed to the left (i.e., negatively skewed). This result indicates a large number of occurrences in the lower value of contact density (left side) and fewer in the higher value of contact density (right side).

### 3.4. Differences in Contact Density of Hand-to-Environment Contact by Participants’ Characteristics

Table 4 shows that the average contact density with Category II items in females (*p* = 0.005), the unemployed (*p* = 0.046), and those with a household size of one member (*p* < 0.001) was significantly higher than that for males, the employed, and those with more than two household members, respectively. For the average contact density with Category III equipment for common use, there were differences according to age (higher for persons over 75 years than those ≤75 years) (*p* = 0.003) and employment status (higher among the unemployed than among the employed) (*p* = 0.050).

## 4. Discussion

In this study, Korean older adults were found to make the most frequent contacts with furniture surfaces, tableware and cooking utensils, and phones, in this order. The average frequency of contact with the environment (number/person) for two hours was 92.2 in Category I, which is mainly used by individuals, and 34.8 in Category III, which is commonly used by several individuals. The frequency of contact with personal equipment or the environment was higher than that of contact with public equipment or the environment. The HEC of older adults mostly showed a higher possibility of self-contamination via HEC with Category I items [5]. However, the contact time (s/contact/person) was 47.7 s for the Category III environment and 14.9 s for the Category I environment. The contact time with public equipment or the environment was about three times longer than that with personal equipment or the environment. Rheinbaben et al. [6] reported that 14 people were contaminated within just 15 s of indirect contact through a door handle contaminated with viruses in a home environment. In the present study, the possibility of pathogen transmission in older adults was very high, over three times that reported in the previous study [6]. The latest coronavirus infection-19 (COVID-19) pathogen, severe respiratory syndrome coronavirus 2 (SARS-CoV-2), survived up to one day on wood or cloth, which are materials found in furniture that the elderly often encounter, and survived for four days on glass surfaces and seven days on plastic or stainless steel surfaces, which are the materials of tableware and cooking utensils [20].

Among the equipment, contact was made most often with furniture surface at 25% and mobile phones at 5.3%. In a 2017 study involving younger age groups [11], contact with telephones, including mobile phones, was the most frequently observed environmental contact, followed by furniture and computers. The average age of the subjects of this study was 75 years, which is higher than the average age (41 years) of the subjects in the 2017 study [11]. The older adults are shown to be more likely to come into contact with furniture than with the phone compared to the younger people who use the phone more frequently. Dry surface contamination [9,21] during the MERS outbreak, as in the case of the recent COVID-19 and SARS-CoV-2 PCR epidemics, was positive on the keyboard surface and the phone surface in the office areas of medical institutions [22]. Compared to personal equipment, commonly used equipment can be more easily contaminated with various microorganisms, and the possibility of cross-contamination among the older adults can be very high. Therefore, it is necessary to educate older adults to avoid unnecessary hand contact with commonly used environmental surfaces and Category III equipment and regularly practice hand hygiene, especially before and after HEC with Category III items. Commonly used environmental surfaces and Category III equipment in the senior welfare centers should be cleaned and disinfected regularly and thoroughly. More specific and detailed cleaning and disinfection guidelines for Category III items that older adults make frequent contact with must be prepared. Hand hygiene products need to be provided at every location of Category III. In spite of the frequency and duration of HEC with Category II showing lower figures, there may still be the risk of cross-contamination, and so unnecessary sharing of personal equipment with others should be avoided. Hand hygiene should be strongly recommended to the older adults to prevent self-contamination via HEC with Category I and cross-contamination via HEC with Categories II and III along with environmental hygiene [23].

In this study, in the case of Category II equipment used for occasional common use, females, unemployed groups, and those who were the only family member had higher contact density than did males, the employed, and those with two or more family members, respectively. In the case of Category III equipment used for public common use, the group over 75 years old and the unemployed group had higher contact density than did the group under 75 years old and the employed, respectively. These findings in this HEC study were contrary to the results of social contact studies that men, young adults, and persons with multi-sized households had higher social contacts than did women, older adults, and those with small-sized households, respectively [24]. Socially active groups of men, the employed, younger adults, and those from large-sized households may be relatively exposed frequently to the risk and precautions of commonly used environment contacts and may be aware of the risk of infection from the environmental surface, thus consciously avoiding contact with equipment used for occasional common use and public use. When awareness of the preventive hygiene rules was high, the number of contacts was small [11]. However, this result needs further investigation owing to the variations of HEC pattern caused by characteristics such as sex and employment status. The results of this study are similar to the result of high contact density in groups of women and unemployed people in the 2017 study, in which younger subjects participated [11]. In the case of those 75 years of age or older, the contact density with common equipment is high for males, the unemployed, or a single family member, and so there are limitations to generalize these results for 30 participants.

Further research will be needed to achieve better understanding. Figure 1 shows that although high contact density is a small number for older adults, there may be a high possibility of becoming a super spreader during an infectious outbreak. This figure may suggest the risk of microbial transmission via Category III equipment, and so thorough environmental control of Category III equipment in the senior welfare center should be emphasized. However, repeated and larger population studies will be necessary for generalization.

The participants in this study had higher income, higher education, and better employment than do ordinary older adults in Korea [8], and so the results of this study have limitations for generalization to all Korean older adults. Only about 72% of the data of video recordings of contact data were analyzed because the participants’ hand contacts were occasionally hidden or covered by other participants. In the future, for this kind of study, it would be useful to record the personal track of each participant through each video recording and for a larger number of participants. Therefore, the results of this study have some limitations in terms of being generalized, as this is a pilot study of a relatively small population of 30 participants.

Nevertheless, this study is the first to examine the frequency and characteristics of HEC of older adults, who are a risk group for infection, as evidence of hand hygiene after HEC, cleaning, and disinfection of environments commonly used in senior welfare centers. This study also presented criteria for classifying environmental equipment according to the risk of contact contamination as a means of infection transmission as well as indices, such as contact density, to measure contact contamination strength in daily life in senior welfare centers among older adults. This will be useful for assessing exposure risk [12] and quantifying transmission [25]. In addition, the study is significant in that it presents basic data to classify high-risk older adults in daily life by revealing that the contact density of older adults differs according to sex, age, occupational status, and number of family members, though it has limitations. It is necessary to increase the number of subjects to derive representative results in future studies.

## 5. Conclusions

This study examined the frequency and characteristics of HEC among Korean older adults during indoor activities in senior welfare centers. Korean older adults made the most frequent contact with furniture surfaces and showed high contact density with Category III equipment. Therefore, it is necessary to practice frequent sanitization of surfaces and hands for commonly used Category III items and regularly practice hand hygiene before and after HEC to prevent self-contamination via Category I items and cross-contamination via Categories II and III items. Commonly used environmental equipment of Category III in the senior welfare centers should be cleaned and disinfected regularly and thoroughly.

## Figures and Tables

**Table 1:** General characteristics of participants (*N* = 30).

Variables	Categories	*N* (%)	M ± SD
Age	66–75 years	15 (50.0)	
	76–85 years	15 (50.0)	
			75.0 ± 4.9
Sex	Male	15 (50.0)	
	Female	15 (50.0)	
Household size	1	12 (40.0)	
	≥2	18 (60.0)	
Education	≤Middle school	16 (53.3)	
	≥High school	14 (46.7)	
Employment	Employed	19 (63.3)	
	Unemployed	11 (36.7)	
Household income (10,000 Korean won)	<200	17 (56.7)	
	≥200	13 (43.3)	

**Table 2:** Contact frequency for equipment items observed to have the maximum hand-to-environment contacts (2-hours observation time, *N* = 30).

Rank	Equipment	*n* (%)	Contact Frequency (Number/Person)		
			Mean (SD)	Median	(Min, Max)
1	Furniture surface	938(25.0%)	31.2(31.5)	25.5	(0.0, 161.0)
2	Tableware and cooking utensils	202(5.4%)	6.7(15.5)	0	(0.0, 51.0)
3	Phone (mobile phone)	200(5.3%)	6.7(11.1)	3	(0.0, 55.0)
4	Door handle	5(0.1%)	0.2(0.9)	0	(0.0, 5.0)
5	Equipment, excluding equipment of ranking 1–4	2, 408(64.2%)	80.3(50.9)	72	(6.0, 234.0)

**Table 3:** Descriptive statistics of hand to environment contact by category for risk of contact transmission (2-hours observation time, *n* = 3,930, *N* = 30 persons).

Contact Indicators	Classification of Equipment	*N* (%)	Mean (SD)	Median (1Q: 3Q)	(Min, Max)
Contact frequency (number/person)					
	Category I	2766 (70.4)	92.2 (53.1)	74.0 (51.3: 127.0)	(17.0, 197.0)
	Category II	120 (3.1)	4.0 (5.7)	1.5 (0.0: 6.3)	(0.0, 25.0)
	Category III	1044 (26.6)	34.8 (33.0)	27.5 (10.0: 50.0)	(0.0, 157.0)
Contact duration (sec/contact/person)					
	Category I	2766 (70.4)	14.9 (12.1)	9.4 (7.6: 19.0)	(2.6, 45.0)
	Category II	120 (3.1)	10.0 (9.1)	8.6 (3.1: 11.7)	(20.0, 33.0)
	Category III	1044 (26.6)	47.7 (193.6)	9.6 (7.6: 14.4)	(2.5, 1053.7)
Sum of contact duration (min/person)					
	Category I	2766 (70.4)	22.7 (19.5)	16.4 (8.7: 32.5)	(1.3, 71.9)
	Category II	120 (3.1)	1.0 (1.0)	0.6 (0.1: 1.4)	(0.0, 3.8)
	Category III	1044 (26.6)	7.6 (9.9)	5.9 (2.1: 10.4)	(0.3, 53.7)
Contact density * (frequency-duration/min/person)					
	Category I	2766 (70.4)	1341.9 (1675.4)	784.5 (238.1: 1806.0)	(11.6, 7077.2)
	Category II	120 (3.1)	3.1 (6.0)	0.2 (0.1: 3.15)	(0.0, 20.9)
	Category III	1044 (26.6)	266.5 (760.6)	51.7 (11.7: 247.0)	(0.0, 4211.5)

* Contact density was obtained by multiplying contact frequency and sum of contact duration, then divided by the given observation time in minutes. Category I: Equipment used by individuals (personally used equipment); Category II: Equipment mainly used by individuals but occasionally by other persons, and Category III: Equipment used for public use (commonly used equipment).

**Table 4:** Differences in density of hand to environments contact by participants’ characteristics (2-hours observation time, *n* = 3930, *N* = 30 person)**.**

Variables	*N* (%)	Contact Density (Frequency-Duration/Min/Person)		
		Category I Median (1Q: 3Q)	Category II Median (1Q: 3Q)	Category III Median (1Q: 3Q)
Sex				
Male	15 (50.0)	845.6 (222.3: 1631.9)	0.017 (0.0: 0.1)	50.8 (2.79: 333.7)
Female	15 (50.0)	723.4 (243.4: 2104.1)	1.71 (0.075: 13.4)	52.5 (11.9: 126.7)
*p*		0.852	0.005 **	0.683
Age				
66–75	15 (50.0)	560.9 (112.3: 2440.6)	0.04 (0.0: 1.71)	11.9 (1.67: 105.1)
76–85	15 (50.0)	845.6 (318.8: 1631.9)	0.10 (0.0: 4.26)	207.2 (50.8: 333.7)
*p*		0.694	0.612	0.003 **
Employment				
Employed	19 (63.3)	560.9 (179.8: 2440.6)	0.025 (0.0: 1.25)	38.03 (2.80: 112.6)
Unemployed	11 (36.7)	857.9 (318.8: 1706.6)	1.45 (0.075: 7.95)	217.6 (35.9: 361.7)
*p*		0.651	0.046 *	0.050 *
Household income (10,000 Korean won)				
<200	17 (56.7)	387.9 (201.1: 1101.5)	0.117 (0.0: 3.58)	52.5 (6.3: 278.9)
≥200	13 (43.3)	1416.3 (461.0: 2524.7)	0.067 (0.0: 2.68)	38.03 (12.7: 266.7)
*p*		0.117	0.522	0.983
Education				
≤Middle school	16 (53.3)	790.7 (227.6: 1634.0)	0.076 (0.006: 3.65)	68.0 (15.8: 219.9)
≥High school	14(46.7)	772.0 (247.8: 2482.7)	0.029 (0.0: 1.90)	44.4 (9.4: 320.2)
*p*		0.678	0.189	0.647
Household size				
1	12 (40.0)	543.1 (262.2: 1305.9)	3.40 (0.48: 15.30)	108.9 (11.54: 222.5)
≥2	18 (60.0)	973.2 (211.7: 2521.7)	0.008 (0.0: 0.081)	44.7 (9.6: 320.2)
*p*		0.397	<0.001 ***	0.672

The normality test (Shapiro–Wilk) of the residuals for category I, II, III for each variable showed non-normality, so the median value, (1Q: 3Q) and non-parametric Mann–Whitney test were used. * *p* <0.05, ** *p* <0.01, *** *p* <0.001. Category I: Equipment used by individuals (personally used equipment); Category II: Equipment mainly used by individuals but occasionally by other person, and Category III: Equipment used for public use (commonly used equipment).

## Data Availability

Data sharing not applicable.
